# Sporadic Occurrence of Jarcho-Levin Syndrome in an Ivorian Newborn

**DOI:** 10.1155/2013/129625

**Published:** 2013-09-11

**Authors:** Thierry Hervé Odéhouri-Koudou, Jean-baptiste Yaokreh, Samba Tembély, Rufin Kobinan Dick

**Affiliations:** ^1^Service de Chirurgie Pédiatrique, CHU de Yopougon, 21 BP 632 Abidjan 21, Cote D'Ivoire; ^2^Université Félix Houphouët-Boigny, BP V 34 Abidjan, Cote D'Ivoire

## Abstract

We report on an isolated chest-wall asymmetry with imaging findings of multiple vertebral and related rib defects in an Ivorian male newborn. He was born of a healthy and young couple without parental lineage, neither family malformative history nor teratogen exposure. This clinical presentation advocates Jarcho-Levin syndrome, a rare sporadic or familial disorder inherited as autosomal dominant or recessive mode and manifested by extensive vertebral segmentation defects with distinctive rib structural and morphological anomalies. According to our belief, this disorder has not been previously traced in the sub-Saharan African area.

## 1. Introduction

Jarcho-Levin syndrome (JLS) also called spondylocostal dysostosis is a congenital costovertebral disorder, occurring with an estimated incidence of 1/40,000 births [1]. JLS can be sporadic or inherited with either autosomal dominant or recessive trait [2]. Its main clinical signs are asymmetric chest wall, short neck-trunk stature, scoliosis, and multiple radiologic vertebral and related rib defects with mild thoracic restriction [3]. Treatment requires earlier respiratory support and later orthopedic or surgical means. We describe a sporadic occurrence of this disorder in an Ivorian male newborn, which to our knowledge has not been previously reported in sub-Saharan Africa.

## 2. A Case Report

**Figure 1:**
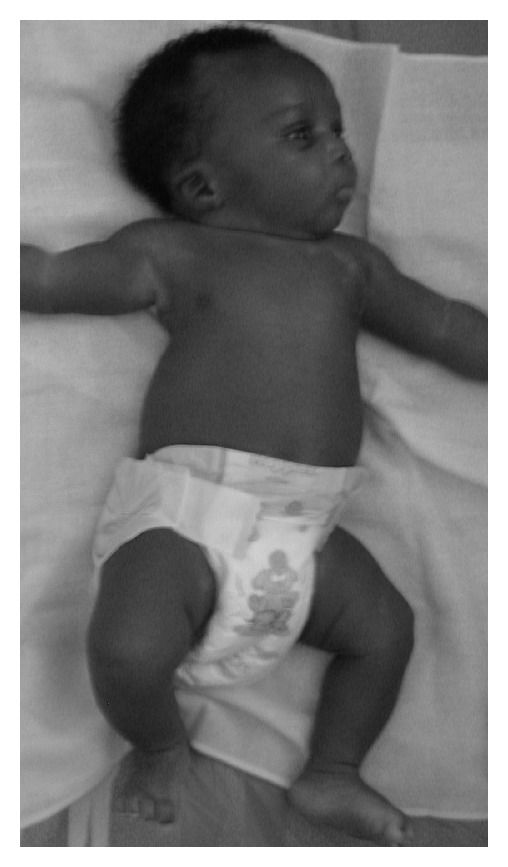
Clinical aspect showing the right-sided basithoracic excavation with the result of an asymmetrical chest-wall deformity.

**Figure 2:**
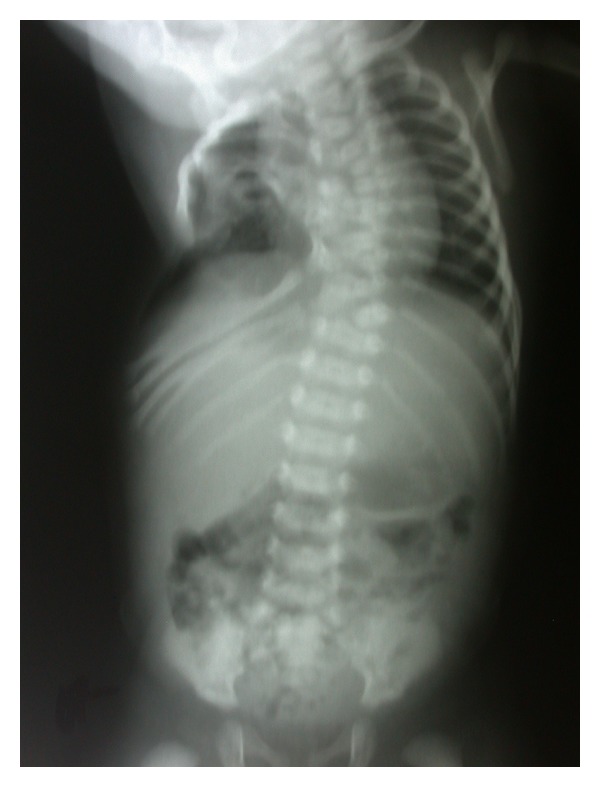
Abdominothoracic AP view showing multiple vertebral bodies defects involving the thoracic spine with related rib abnormalities.

**Figure 3:**
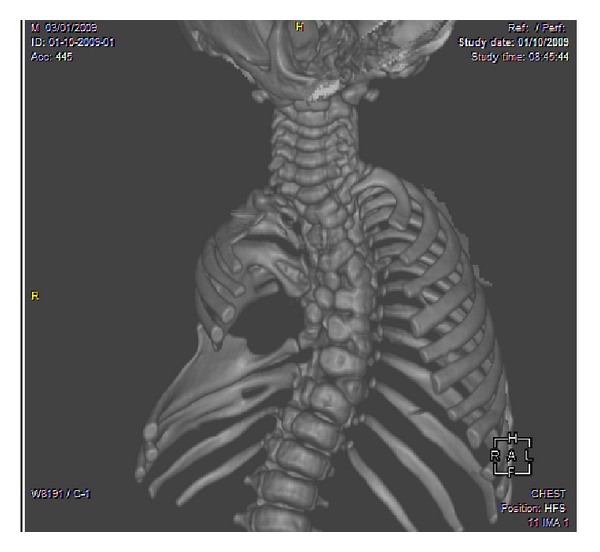
Three-dimensional CT scan AP thoracic image revealing full details of block, fused, and hemivertebrae as well as misaligned, absent, fused, and hypoplastic ribs at the right side and supernumerary ribs at the opposite side.

**Figure 4:**
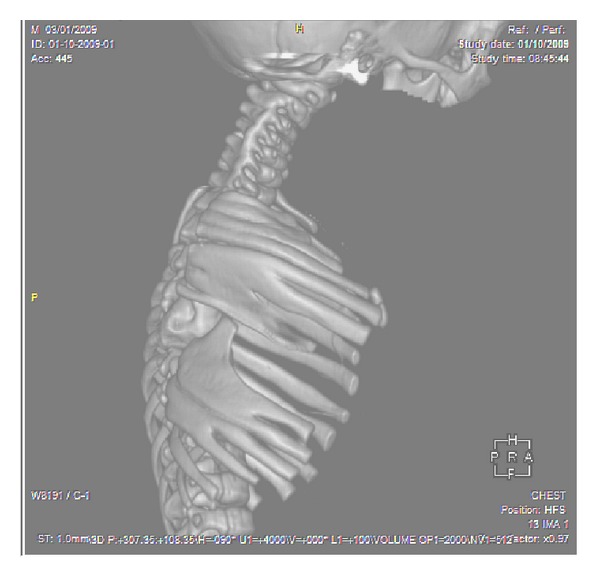
Three-dimensional CT scan lateral thoracic image demonstrating many rib points of fusion originated posteriorly.

A full-term newborn, second of two normal siblings, born of a healthy and nonrelated couple with negative history of familial anomaly and prenatal ultrasound, was born vaginally after an uneventful and drug-free pregnancy. Birth weight was 3100 g, length 48 cm, cranial perimeter 33 cm, and Apgar score 8-9 at 5 minutes. He showed right-sided basithoracic excavation with an asymmetrical chest-wall aspect (Figure 1). X-rays revealed costovertebral anomalies of the thoracic spine (Figure 2) and CT scan full details, including fused or hemivertebrae and misaligned and fused ribs with three absent ribs at the right side and as much supernumerary at the left side (Figures 3 and 4). Abdominal and renal ultrasound found no associated visceral anomaly and chromosomal study karyotype 46, XY. Its clinical course revealed progressive spinal curvature with breathing compromise. At 3 years of age, he was transferred to Children's Hospital of Philadelphia, where he underwent a rib implant surgery with satisfactory outcome.

## 3. Discussion

JLS depicts a panethnic disorder defined by asymmetrical rib defects, varying from absent to fused to overgrown, as well as fused, block, and hemivertebrae with mild breathing restriction, which occurs sporadically rather than as inherited [2, 3]. As we know, few cases have been cited in northern and southern Africa, but none formerly traced in sub-Saharan Africa [4, 5]. The case reported above is consistent with JLS clinical picture. Yet, there has been still controversy about the nosologic delineation and the genetic basis of this complex topic in the literature.

On genetic grounds, most familial cases appear to follow an autosomal recessive mode of inheritance, with linkage to the Delta-like 3 gene, associated with the Notch pathway [2]. In ours, negative family history and nonconsanguinity, as well as unaffected siblings suggest a sporadic occurrence. However, this pattern proved to be poorly documented in the literature compared to the familial ones. In such pattern fitting normal karyotype, as our case, the genetics remain elusive since there is to date no confident mutated case besides the evocation of few candidate genes [2]. So, it is hopeless to offer reliable and accurate genetic counseling to affected families. 

Clinically, neither classical short trunk-neck aspect nor associated visceral anomalies observed in sporadic cases, were seen in ours, agreeing with reportedly heterogeneity of such instances [2, 3]. Thus, our diagnosis was based on the radiographic cardinal signs of SCD. At this point, there has been confusion about the nosologic delineation of this field, mainly with spondylothoracic dysostosis (STD). A recent historical review by Berdon et al. [6] has consistently stressed on the distinctive clinical and imaging features of these two phenotypically similar syndromes. Therefore, SCD must be differed from STD that refers to Lavy-Moseley syndrome, largely linked to Puerto Rican cohorts and typified by the so-characteristic symmetrical posterior rib fusion giving a “fan-like” or “crab-like” radiographic aspect and a grimmer prognosis due to markedly shortened thorax causing severe respiratory insufficiency [7, 8]. Even if SCD is usually diagnosed at birth as in the present case, prenatal ultrasonographic detection should be desirable to allow a better parental psychological preparation [9]. 

No standardized treatment exits for this disorder, but anyhow we could go no further than the prevention of pulmonary infections to avoid respiratory compromise [6]. As in our patient, surgical procedures can be planned depending on evolutional course. The innovative expandable titanium rib implant, still under investigational protocol, only by specialized teams, emerged as a promising new surgical option [10].
